# A closer look to the lung involvement in ANCA-associated vasculitis: alveolar haemorrhage and interstitial lung disease

**DOI:** 10.1007/s10238-025-01756-2

**Published:** 2025-10-31

**Authors:** Mehmet Nur Kaya, Özlem Kılıç, Sedat Yılmaz

**Affiliations:** Rheumatology Department, Gülhane Training and Research Hospital, University of Health Sciences Turkey, Ankara, Turkey

**Keywords:** Antineutrophil cytoplasmic autoantibody, Granulomatosis with polyangiitis, Microscopic polyangiitis, Diffuse alveolar haemorrhage, Interstitial lung disease

## Abstract

Interstitial lung disease (ILD) and alveolar haemorrhage (AH) have been described as possible pulmonary involvements associated with antineutrophil cytoplasmic antibody (ANCA)-associated vasculitis (AAV), mainly granulomatosis with polyangiitis (GPA) and microscopic polyangiitis (MPA). To evaluate the clinical, laboratory and demographic features of ANCA positivity in AAV; in particular, we aimed to determine the criteria predicting ILD involvement and AH. This study was designed as a cross-sectional and retrospective study conducted in a single centre. The studies comprised 83 individuals in total who were routinely followed at a tertiary rheumatology clinic between June 2016 and February 2024. A total of 83 patients consisting of 56 GPA (67.4%), 22 MPA (26.5%) and 5 ANCA-negative (6.1%) cases were included in the study. When adjusted for age and sex in a multivariate regression model, the correlation of AH with C-ANCA was confirmed (OR 4.80; 95% CI 1.29–17.91) (*p* = 0.020). The subsequent multivariate regression analysis reveals that the P-ANCA variable significantly predicts ILD (OR 4.28; 95% CI 1.25–14.65) (*p* = 0.021) when adjusted for age and sex. AH was found to be higher in patients with GPA and in patients with C-ANCA positivity. It was shown that P-ANCA positivity predicted ILD involvement in AAV patients. The widespread ILD involvement observed in P-ANCA-positive patients confirmed usual interstitial pneumonia, but nonspecific interstitial pneumonia was also found to predominate in C-ANCA-positive and ANCA-negative cases.

## Introduction

Antineutrophil cytoplasmic autoantibody (ANCA)-associated vasculitis (AAV) are a heterogeneous group of diseases that include granulomatosis with polyangiitis (GPA), microscopic polyangiitis (MPA) and eosinophilic granulomatosis with polyangiitis (EGPA) [1, 2]. In clinical practice, ANCA can be detected either using antigen-specific enzyme-linked immunosorbent assays (ELISA) for proteinase 3 (PR3) and myeloperoxidase (MPO) or using C-ANCA and P-ANCA indirect immunofluorescence (IIF) testing. While MPO-ANCA and P-ANCA are frequently detected in MPA and EGPA, PR3-ANCA and C-ANCA are more commonly seen in GPA [3]. The diagnosis of AAV is not ruled out by a negative ANCA, nor are these ANCA specificities pathognomonic for any distinct AAV. Diagnosis is usually defined by the presence of a combination of clinical and serological findings and/or histological evidence of necrotizing pauci-immune small-vessel vasculitis [1, 4].

Prodromal symptoms may persist for weeks to months without evidence of specific organ involvement [5]. In GPA and MPA, small blood vessels in almost any organ or tissue can be affected, but the most important and frequent involvement is in the lungs and kidneys, with the exception of the upper respiratory tract involvement characteristic of GPA [6]. Involvement of the airways or pulmonary parenchyma causing, cough, dyspnea, haemoptysis, or pleuritic pain may occur in patients with both GPA or MPA [7]. Different types of pulmonary involvement such as pulmonary nodule, cavitation, infiltration, interstitial lung disease (ILD) and alveolar haemorrhage (AH) have been described in AAV patients that may cause these symptoms [8].

The AAV syndromes GPA and MPA are the most common causes of pulmonary capillaritis leading to AH [9]. AH is a serious manifestation of AAV, is associated with glomerulonephritis and often leads to respiratory failure requiring ventilatory support in the intensive care unit [10].

ILD is a rare condition that is well described in its idiopathic forms such as idiopathic pulmonary fibrosis (IPF), but is also known as a common complication in immune-mediated rheumatic diseases such as systemic sclerosis, rheumatoid arthritis and Sjogren syndrome [11, 12]. The majority of our information regarding ILD during AAV comes from case series, which indicate that ILD typically affects older patients who have detectable circulating MPO-ANCA. The most prevalent pattern observed on high-resolution computed tomography (HRCT) is usual interstitial pneumonia (UIP).

The objective of this investigation was to evaluate clinical, laboratory, and demographic characteristics in relation to ANCA positive in AAV; especially, we sought to identify the criteria that are predictive of ILD involvement and AH.

## Methods

### Study design

This study was designed as a cross-sectional and retrospective study conducted in a single centre. Eighty-nine patients with GPA and MPA who were initially diagnosed were included in the study, but six patients who did not have regular follow-up were excluded from the study. The study comprised 83 individuals in total who were routinely monitored at a tertiary rheumatology clinic between June 2016 and February 2024. The present study received ethical approval from the local ethics committee, in accordance with the principles outlined in the Declaration of Helsinki (Date: 19.03.2024 and Decision number: 2024/137).

### Patients and data collection

We applied the criteria retrospectively to patients followed up with a diagnosis of AAV between 2016 and 2022. Patients diagnosed with AAV in 2022 and later were evaluated using American College of Rheumatology (ACR)/European Alliance of Associations for Rheumatology (EULAR) classification criteria [13, 14]. Patients over 18 years of age who met the diagnostic criteria were included in the study. The patients included in the study were diagnosed with both new-onset and recurrent AAV. Patients with diseases that could cause parenchymal damage in the lung (emphysema, idiopathic pulmonary fibrosis, sarcoidosis, etc.), infectious diseases of the lung, malignancies, other systemic inflammatory rheumatic diseases and overlap syndrome with other autoimmune disease were not included in the study.

Demographic data, clinical findings, laboratory results, imaging findings and treatment methods of the patients were obtained from the patient files. Demographic data and clinical findings of the patients were evaluated as gender, age of disease onset, age at the onset of treatment, smoking, organ and system involvement and treatment status. The laboratory parameters assessed were; C-reactive protein (CRP) (0–5 mg/L), erythrocyte sedimentation rate (ESR) (5–20 mm/h). Serum ANCA positivity was tested by IIF on ethanol-fixed neutrophils [15].

Assessment of AAV lung involvement was performed by a multidisciplinary evaluation involving at least one rheumatologist and pulmonologist using HRCT and pulmonary function testing (PFT). The last HRCT and PFT available at the time of recording was taken into account to determine the radiological pattern. PFTs were performed according to standard methods [16, 17]. PFT results were expressed as a percentage of the predicted value of each parameter and corrected for age, sex and height. Respiratory function was assessed by forced vital capacity (FVC). The single-breath diffusion capacity of the lung for carbon monoxide (DLCO) was used to assess gas transfer. In patients with suspected GPA or MPA, bronchoalveolar lavage (BAL) performed during flexible bronchoscopy may be helpful in AH, but clinical and serological findings play an important role in determining a specific aetiology.

### Statistical analysis

The statistical analyses were conducted using version 28 of Statistical Package for Social Sciences (Armonk, NY: IBM Corp) program for Windows. Kolmogorov–Smirnov and Shapiro–Wilk’s tests were utilized to assess whether the variables were normally distributed. Normally distributed variables were expressed as mean ± standard deviation (SD), skewed variables as median (interquartile range-IQR), and categorical variables as number (n) and percentage (%). Patients' demographic clinical and laboratory characteristics were compared between these two groups using the Chi-Square test or Fisher’s exact test (when Chi-square test assumptions do not hold) for categorical data, the Mann–Whitney U test for non-normally distributed continuous data, and independent T test for normally distributed continuous data. A forward stepwise multivariable regression model was developed using factors that had significant associations with ILD and AH in univariate analysis. Statistical significance was determined by accepting p-values that were less than 0.05.

## Results

Eighty three patients with AAV vasculitis were included in the study. The mean age of the patients was 47.6 (14.1) years and 46 (55.4%) of them were male. The mean follow-up period of the patients was 4.8 (2.1) years. Fifty six patients were followed up with a diagnosis of GPA (67.4%), 22 with MPA (26.5%) and 5 (6.1%) with ANCA-negative AAV. The most common systemic involvement at diagnosis was pulmonary involvement in 54 (65.1%) patients, and the second most common involvement was renal involvement 29 (34.9%). During clinical follow-up, the most common systemic involvements were determined to be pulmonary 63 (75.9%) and renal involvement 53 (63.9%), respectively. All patients received glucocorticoid treatment, and other immunosuppressive treatments were given alone or in combination. Details regarding demographic, serological, clinical characteristics and treatment status of AAV patients are summarized in Table 1.

**Table 1:** Demographic, clinical and laboratory characteristics of the patients

Variables	Values
Male, *n* (%)	46 (55.4)
Age at onset, mean (SD)	47.6 (14.1)
Age at the onset of treatment, years, mean (SD)	48.9 (13.9)
Follow-up, years, mean (SD)	4.8 (2.1)
Ever smokers, *n*%	24 (28.9)
C-ANCA, *n*%	56 (67.4)
P-ANCA, *n*%	22 (26.5)
ANCA-negative	5 (6.3)
CRP (mg/L), median (IQR) (first clinic time)	113.2 (59.1)
ESR (mm/h), median (IQR) (first clinic time)	85.4 (24.5)
*First clinical appearance (more results possible), n%*	
Pulmonary involvement	54 (65.1)
Renal involvement	29 (34.9)
ENT involvement	22 (26.5)
Cutaneous involvement	14 (16.9)
Neurologic involvement	5 (6.3)
Other	14 (16.9)
*Clinical symptoms (more results possible), n%*	
Pulmonary involvement	63 (75.9)
Renal involvement	53 (63.9)
ENT involvement	27 (32.5)
Cutaneous involvement	22 (26.5)
Neurologic involvement	19 (22.9)
Arthritis	17 (20.5)
Other	12 (5.2)
*Treatments, n%*	
Glucocorticoids	83 (100)
Cyclophosphamide	47 (56.6)
Rituximab	38 (45.8)
Azathioprine	41 (49.4)
Methotrexate	21 (25.3)
Mycophenolate mofetil	15 (18.1)

*SD* standard deviation, *IQR* inter-quartile range, *ANCA* antineutrophil cytoplasmic antibodies, *CRP* C-reactive protein, *ESR* erythrocyte sedimentation rate, *ENT* ear, nose and throat

Thirty-four (60.7%) of the C-ANCA positive and 10 (45.5%) of the P-ANCA positive were male. In the P-ANCA-positive group, the age of onset and the age of treatment initiation were found to be significantly higher than the other group, respectively (*p* < 0.034, *p* < 0.038). When evaluated in terms of systemic involvement, the number of patients with pulmonary involvement, renal involvement and ear, nose and throat (ENT) involvement was found to be significantly higher in the C-ANCA-positive group than in the P-ANCA-positive group, respectively (*p* < 0.039, *p* < 0.012, *p* < 0.040). AH and ILD were found to be considerably more prevalent in the C-ANCA-positive group when pulmonary involvement subgroups were analysed (*p* < 0.040, *p* < 0.016). Other differences between the groups are given in Table 2. Among the five ANCA-negative AAV patients followed up, one (20%) had AH and two (40%) had ILD. The general characteristics of the ANCA-negative patients are summarized in Table 3.

**Table 2:** Demographic, clinical and serological characteristics of patients according to ANCA

Variables	C-ANCA (*n* = 56)	P-ANCA (*n* = 22)	*p*
Female, *n* (%)	22 (39.3)	12 (54.5)	0.311
Male, *n* (%)	34 (60.7)	10 (45.5)	0.221
Age at onset, mean (SD)	45.1 (14.1)	52.7 (14.1)	0.034
Age at the onset of treatment, years, mean (SD)	46.5 (13.9)	53.8 (13.7)	0.038
Follow-up, years, mean (SD)	5.1 (2.1)	5.7 (1.9)	0.067
*Clinical symptoms (more results possible), n%*			
Renal involvement	40 (71.4)	9 (40.9)	0.012
ENT involvement	21 (37.5)	3 (13.6)	0.040
Cutaneous involvement	15 (26.8)	7 (31.8)	0.657
Neurologic involvement	10 (17.9)	7 (31.8)	0.179
Arthritis	13 (23.2)	3 (13.7)	0.346
Pulmonary involvement	44 (78.6)	12 (54.5)	0.039
Nodule	24 (42.9)	3 (13.6)	0.015
Cavitation	13 (23.2)	1 (4.5)	0.032
AH	21 (37.5)	3 (13.6)	0.040
Infiltration	22 (39.3)	3 (13.6)	0.029
Asthma	2 (3.6)	10 (43.4)	< 0.001
ILD	6 (10.7)	7 (31.8)	0.016
*ILD pattern, n%*			
UIP	2 (3.6)	5 (22.7)	0.036
NSIP	4 (7.1)	2 (9.1)	0.344
FVC %, median (IQR)	87.5 (9.5)	80.5 (10.5)	0.452
DLCO %, median (IQR)	68 (13)	57.5 (5.7)	0.224
CRP (mg/dL), median (IQR) (first clinic time)	113.4 (62.6)	112.9 (50.1)	0.974
ESR (mm/h), median (IQR) (first clinic time)	85.3 (26.4)	85.6 (19.6)	0.970

*SD* standard deviation, *IQR* inter-quartile range, *ANCA* antineutrophil cytoplasmic antibodies, *ENT* ear, nose and throat, *AH* alveolar haemorrhage, *ILD* interstitial lung disease, *UIP* usual interstitial pneumonia, *NSIP* nonspecific interstitial pneumonia, *FVC* forced vital capacity, *DLCO* diffusing capacity of the lung for carbon monoxide, *CRP* C-reactive protein, *ESR* erythrocyte sedimentation rate

**Table 3:** Characterization of pulmonary involvement in ANCA-negative AAV patients

Case	Diagnosis	Demographic, clinical and laboratory findings at diagnosis
Patient 1	GPA	Lung nodules, AH, arthritis, ENT involvement*, multiple mononeuritis**, constitutional symptoms
Patient 2	GPA	Lung nodules and cavitations, ILD, petechiae and purpura, ENT involvement*
Patient 3	GPA	Lung nodules and infiltrates, ENT involvement*, constitutional symptoms, skin vasculitis**
Patient 4	GPA	Renal involvement, constitutional symptoms, ENT involvement*, petechiae and purpura
Patient 5	MPA	Asthma, lung infiltrates, ILD, peripheral neuropathy, constitutional symptoms, skin vasculitis**

*Ear, nose, throat, including otitis media, bloody nasal discharge; **confirmed by biopsy ANCA: Antineutrophil cytoplasmic antibodies; *AAV* ANCA-associated vasculitis, *GPA* granulomatosis with polyangiitis, *MPA* microscopic polyangiitis, *AH* alveolar haemorrhage, *ILD* interstitial lung disease, *ENT* ear, nose and throat

Univariate regression analysis was performed to evaluate possible correlations between AH on HRCT and AAV clinico-serological features, and a significant association was demonstrated with C-ANCA and the occurrence of AH (OR 4.80; 95% CI 1.29–17.91) (*p* = 0.020). Based on these results, a multivariate regression model was also performed. The analysis confirmed the correlation of AH with C-ANCA (OR 4.80; 95% CI 1.29–17.91) (*p* = 0.020) when adjusted for age and sex (Table 4). The results of univariate regression analysis showed a significant relationship between ILD and P-ANCA (OR 4.28; 95% CI 1.25–14.65) (*p* = 0.021). The subsequent multivariate regression analysis reveals that the P-ANCA variable significantly predicts ILD (OR 4.28; 95% CI 1.25–14.65) (*p* = 0.021) when adjusted for age and sex (Table 5).

**Table 4:** Univariate and multivariate regression analysis examining the association between AAV with AH

Covariate	Univariate			Multivariate		
	OR	*p*	95% CI (lower–upper)	OR	*p*	95% CI (Lower–Upper)
C-ANCA	4.80	0.020	1.29–17.91	4.80	0.020	1.29–17.91
P-ANCA	1.11	0.850	0.39–3.17			
Smoke	0.71	0.584	0.21–2.44			
Male	0.77	0.657	0.24–2.51			
Age at onset	1.01	0.616	0.97–1.05			
GPA	3.59	0.059	0.96–13.49			

*OR* odds ratio, *CI* confidence interval, *ANCA* antineutrophil cytoplasmic antibodies, *AAV* ANCA-associated vasculitis, *AH* alveolar haemorrhage, *GPA* granulomatosis with polyangiitis

**Table 5:** Univariate and multivariate regression analysis examining the association between AAV with ILD

Covariate	Univariate			Multivariate		
	OR	*p*	95% CI (lower–upper)	OR	*p*	95% CI (lower–upper)
C-ANCA	0.35	0.082	0.11–1.15			
P-ANCA	4.28	0.021	1.25–14.65	4.28	0.021	1.25–14.65
Smoking	1.20	0.801	0.29–4.95			
Male	0.64	0.466	0.19–2.11			
Age at onset	1.01	0.251	0.97–1.05			
MPA	3.94	0.028	1.16–13.41			

*OR* odds ratio, *CI* confidence interval, *ILD* interstitial lung disease, *ANCA* antineutrophil cytoplasmic antibodies, *AAV* ANCA-associated vasculitis, *MPA* microscopic polyangiitis

## Discussion

In this article, demographic, clinical, laboratory and radiological characteristics of AAV patients according to ANCA testing were compared in a cross-sectional retrospective study. According to our findings, C-ANCA predicted the presence of AH in AAV patients, which was diagnosed by clinical, imaging, and BAL data. The P-ANCA predicted the ILD that was found by HRCT. Patients who tested positive for P-ANCA also showed a higher frequency of UIP pattern.

The French Vasculitis Study Group recently reported findings in 80 patients with AH resulting from GPA or MPA from their database. Most of the clinical information was obtained by questionnaires sent to the treating physician, and AH was not confirmed by BAL in all cases [18]. Among the patients in this study, 49 (61.3%) were GPA, 21 (26.3%) were MPA and 8 (10%) were Churg-Strauss syndrome (CSS). In a different investigation, 53 individuals with GPA- or MPA-related AH were gathered over a 21-year period at two locations in the Czech Republic and the United Kingdom; only one-third of these patients had bronchoscopy confirmation of AH. Of the patients with AAV included in this study, 37 (69.8%) were diagnosed with GPA and 16 (30.2%) were diagnosed with MPA [10]. In a study conducted by Cartin-Ceba et al. on a large cohort of 73 patients with AH diagnosed with AAV, 60% of the patients were found to have GPA and 40% were found to have MPA. The study also found that respiratory failure and the requirement for plasma exchange were more prevalent in patients with PR3-ANCA positivity [19]. In addition to a high prevalence of concomitant active renal disease, our population consisted primarily of patients with GPA rather than MPA. In our study, when smoking, age, gender, GPA patients and serological parameters were evaluated, although GPA patients were not found to be statistically significant, it was found to be more significant than other parameters. In other studies where AH was seen in patients with AAV, AH was found to be higher in patients with GPA and patients with C-ANCA positivity, but no further analyses were performed to show its superiority over other parameters. In multivariate regression analysis, C-ANCA was found to predict AH.

Over the last few years, the number of publications reporting the association between ILD and AAV has been increasing. In a study conducted by Comarmond et al. [20] in 2014, a population of 49 patients with AAV-associated ILD was described. ANCA MPO was detected in 88% of the cases, while UIP was the most frequently detected radiological pattern in 43% of the patients. A large AAV-ILD cohort was described in a study by Maillet et al. [21] of 62 AAV-ILD patients, 53 with MPA and 9 with GPA. The majority of patients (63%) showed a UIP pattern, while only 24 (35%) patients had nonspecific interstitial pneumonia (NSIP) pattern [21]. A large Italian cohort included 95 consecutive patients with AAV-ILD, of whom 56 were affected by MPA (58.9%) and 39 by GPA (41.1%). NSIP was the most frequently detected ILD pattern observed in 60.9% of cases in C-ANCA patients, followed by UIP pattern observed mainly in P-ANCA patients [22]. Our data suggest that P-ANCA positivity predicts ILD involvement in AAV patients. While UIP was more prevalent in P-ANCA-positive patients, NSIP was more prevalent in C-ANCA-positive and ANCA-negative patients.

Our study has several limitations, but it also has some strengths. First, because data were collected retrospectively at disease onset and diagnosis, incomplete data sets may have influenced the findings. Other limitations include possible referral bias; patients in this cohort may have represented patients with more severe ILD and AH, while asymptomatic or mildly symptomatic patients may have been less frequently identified and included in the study. The evaluation of specific ELISA tests for the presence of antibodies directed against PR3 and MPO was not possible due to the unavailability of these assays at the hospital. Finally, the lack of a central reading of HRCTs may represent a bias in HRCT pattern definition, but, on the other hand, they had thoracic radiologists with high expertise in parenchymal lung diseases. Furthermore, patients were evaluated uniformly with a standardized data collection.

## Conclusion

As a result, it was determined that AH was higher in patients with GPA and patients with C-ANCA positivity, while P-ANCA positivity was shown to predict ILD involvement in AAV patients. ILD involvement observed in P-ANCA-positive patients was detected as UIP, but NSIP was also determined to be dominant in C-ANCA-positive and ANCA-negative cases. Further prospective studies with larger and more homogeneous populations are needed to better define the prevalence and evolution of ILD and AH in AAV, its clinical phenotype and possibly a better therapeutic approach.

## Data Availability

No datasets were generated or analysed during the current study.
